# Differential spatiotemporal dynamics of cell wall-degrading enzymes underlie pathogenicity variation in two *Alternaria* species causing kiwifruit soft rot

**DOI:** 10.7717/peerj.21223

**Published:** 2026-04-28

**Authors:** Li-Zhen Ling, Si-Han Tai, Hui Teng, Shu-Dong Zhang

**Affiliations:** Key Laboratory for Specialty Agricultural Germplasm Resources Development and Utilization of Guizhou Province, Liupanshui Normal University, Liupanshui, Guizhou, China

**Keywords:** Kiwifruit soft rot, *Alternaria*, CWDEs, Pathogenic difference

## Abstract

Kiwifruit soft rot caused by fungal pathogens can result in substantial postharvest losses. While *Alternaria alternata* and *A. tenuissima* have been implicated in this disease, their pathogenic mechanisms remain poorly characterized. This study investigated the spatiotemporal dynamics of cell wall-degrading enzyme (CWDE) activities during infection of kiwifruit by *A. alternata* (strain P1-1W) and *A. tenuissima* (strain P1-2W). Using a zonal sampling approach (healthy, marginal, and lesioned tissues) over 0–6 days post-inoculation (dpi), we analyzed the activities of six CWDEs: polygalacturonase (PG), polymethylgalacturonase (PMG), PG trans-eliminase (PGTE), PMG trans-eliminase (PMTE), cellulase (Cx), and β-glucosidase (β-Glu). Our results demonstrated that infection time, rather than spatial proximity to lesions, was the primary determinant of CWDE activity profiles for both pathogens. Despite this shared temporal regulation, the two species exhibited distinct enzymatic strategies. *A. alternata* deployed a PMG-centric, biphasic infection strategy, characterized by early and sustained PMG induction (peaking at 37.08 U/mL at 4 dpi), with significant contributions from Cx, β-Glu and PG at later stages. In contrast, *A. tenuissima* adopted a cellulase-dominant strategy, where Cx and β-Glu were the principal drivers of tissue maceration (exhibiting sustained elevation from 3–6 dpi). Two pectinases (PG and PMG) played transient, early roles. Activities of PGTE and PMTE were negligible for both species. These findings reveal that closely related *Alternaria* species employ divergent, temporally programmed CWDE arsenals to infect kiwifruit, providing new insights into their pathogenic mechanisms.

## Introduction

Kiwifruit (*Actinidia* spp.) is a globally significant horticultural crop, valued for its exceptional nutritional profile, which includes remarkably high vitamin C content, dietary fiber, and beneficial phytochemicals such as flavonoids (Liu et al., 2019; Ma et al., 2017; Nishiyama, 2007; Sivakumaran et al., 2018). This high commercial and nutritional value, however, is severely compromised by postharvest diseases. Among these, soft rot disease stands out as a major constraint, causing substantial economic losses estimated at 20–50% annually in leading production regions like China and New Zealand (Huang, 2014; Nishiyama, 2007). These losses not only impact economic returns but also contribute to significant food waste, underscoring the urgent need for effective management strategies. The etiology of kiwifruit soft rot disease is complex, involving a consortium of fungal pathogens. Notably, *Botryosphaeria dothidea* and species within the genus *Diaporthe* spp. have been consistently identified as primary causal agents, playing a pivotal role in the development of postharvest decay (Díaz et al., 2014; Zhou et al., 2015).

The genus *Alternaria* represents one of the most successful and ubiquitous groups of plant pathogens, comprising species with over 4,000 documented host associations (Florea & Puia, 2020). These pathogens typically infect plant leaves, flowers, fruits, roots, stems, and seedlings, causing various types of lesions (Florea & Puia, 2020). *Alternaria* species have also been identified as causal agents of kiwifruit soft rot. Among them, *A. alternata* is widely recognized as an important rot pathogen in kiwifruit (Li et al., 2019; Ling et al., 2023).  In contrast, *A. tenuissima* has been previously reported to cause brown spot disease on kiwifruit foliage and fruit scab in China (Li et al., 2019; Ma et al., 2020). Notably, our group first reported that *A. tenuissima* (strain P1-2W) is capable of infecting kiwifruit during cold storage, leading to soft rot development (Ling et al., 2023). In the same study, we also isolated *A. alternata* (strain P1-1W) from decayed ’Hongyang’ kiwifruit. Despite these findings, the mechanisms underlying their pathogenicity remain poorly understood.

This ecological success is underpinned by a multifactorial pathogenic strategy. A central pillar of this strategy is the secretion of an array of cell wall-degrading enzymes (CWDEs). These include polygalacturonases, pectin lyases, and cellulases that systematically dismantle the plant’s structural integrity. Specifically, they depolymerize key polysaccharide components such as pectin and cellulose in a process that leads to tissue maceration and the subsequent release of nutrients (Florea & Puia, 2020; Ma et al., 2019). Beyond their direct degradative function, these CWDEs have gained recognition for their dual role in plant-pathogen interactions. Specifically, oligosaccharide fragments released by CWDE activity, as well as some enzymes themselves, can function as pathogen-associated molecular patterns (PAMPs) (De Lorenzo & Cervone, 2022; Tariqjaveed et al., 2021; Wang et al., 2024a; Wang et al., 2024b). These PAMPs are perceived by host membrane-localized pattern recognition receptors (PRRs). This recognition event triggerse the first layer of induced defense, known as pattern-triggered immunity (PTI) (Hückelhoven, 2007). PTI activation leads to canonical defense responses that include rapid ion flux (*e.g.*, Ca^2^^+^ influx), a burst of reactive oxygen species (ROS), activation of mitogen-activated protein kinase (MAPK) cascades, and extensive transcriptional reprogramming (De Lorenzo & Cervone, 2022; Meng & Zhang, 2013; Naveed et al., 2024). Therefore, the outcome of infection is a delicate balance between the pathogen’s enzymatic aggression and the host’s capacity to detect and respond to these invasive signals.

Our previous studies have confirmed the importance of pectinases and cellulases in the pathogenicity of *Diaporthe* species causing kiwifruit soft rot (Ling et al., 2024a; Ling et al., 2024b). The involvement of CWDEs in *Alternaria* pathogenicity has been recognized (Wang et al., 2024a). While the genome annotation of *Alternaria* species has identified a large repertoire of genes encoding carbohydrate-active enzymes (CAZymes) (Chen et al., 2024; Huang et al., 2021). How these enzymes are deployed across the infection interface—and whether their dynamic patterns differ between *Alternaria* species—have not been characterized. In this study, we employed a zonal sampling approach to dissect the spatiotemporal dynamics of pectinolytic and cellulolytic activities during infection by two *Alternaria* species, providing the first detailed mapping of CWDEs distribution across healthy, marginal, and lesioned tissues in the kiwifruit-*Alternaria* pathosystem. This study will elucidate the enzymatic strategies employed by different *Alternaria* species during kiwifruit infection but also provide insights into the contribution of CWDEs to their pathogenic potential.

## Materials and Methods

### Plant material and fungal pathogens

Mature, commercially ripe ‘Hongyang’ kiwifruit (*Actinidia chinensis*) were selected based on uniformity of size and the absence of visual defects or mechanical injury. The two fungal pathogens used in this study, *Alternaria alternata* (strain P1-1W) and *Alternaria tenuissima* (strain P1-2W), were previously isolated from decaying ‘Hongyang’ kiwifruit and characterized in our laboratory (Ling et al., 2023). For inoculum preparation, both strains were cultured on Potato Dextrose Agar (PDA) medium and incubated at 25 ^∘^C in darkness for 3 days.

### Fruit inoculation and tissue sampling

Fruits were surface-sterilized by sequential immersion in 5% (v/v) sodium hypochlorite solution for 2 minutes followed by 75% (v/v) ethanol for 2 minutes and then rinsed four times with sterile distilled water and air-drying. Each fruit was wounded with a sterile needle to create five evenly distributed puncture sites. A mycelial plug (five mm in diameter) from a 3-day-old culture was then placed onto each wound site. Control fruits were inoculated with sterile PDA agar plugs of the same size. All inoculated fruits were placed in sterile, humidified containers and incubated at 28 ^∘^C to promote disease development.

Tissue samples were collected at 0, 0.5, 1, 2, 3, 4, 5, and 6 days post-inoculation (dpi). Using a sterile scalpel, each fruit were precisely dissected into three distinct zones: healthy tissue (1–2 cm from any lesion), lesioned tissue (the necrotic and macerated area), and the marginal tissue (the advancing interfacial interface of lesioned tissues) (Fig. S1). Samples from each zone were immediately flash-frozen in liquid nitrogen and stored at −80 ^∘^C until enzyme extraction. Each treatment (pathogen × time point × tissue zone) consisted of three biological replicates, with each biological replicate consisted of pooled tissue collected from six inoculation sites on a single fruit.

### Preparation of crude enzyme extracts

Frozen tissue samples were ground to a fine powder in liquid nitrogen using a sterilized mortar and pestle. The powder was homogenized in pre-chilled extraction buffer (1 M NaCl) at a ratio of 1:4 (w/v), which contained 10 mmol/L EDTA and 5 g/L PVP (pH of 7.4, using 0.01 mol L^−1^ NaOH). The homogenate was centrifuged at 10,000× g for 15 minutes at 4 ^∘^C. The resulting supernatant was collected as the crude enzyme extract and kept on ice for immediate use in enzyme activity assays.

### Assays for cell wall-degrading enzyme activities

The activities of key cell wall-degrading enzymes were determined spectrophotometrically. The activities of polygalacturonase (PG), polymethylgalacturonase (PMG), cellulase (Cx), and β-glucosidase (β-Glu) were quantified using the 3,5-dinitrosalicylic acid (DNS) method to measure the release of reducing sugars from their respective substrates, as described previously (Ling et al., 2024a). The activities of PG trans-eliminase (PGTE) and PMG trans-eliminase (PMTE) were assayed according to established protocols (Ling et al., 2024a). Enzyme activity was expressed in specific units (*e.g.*, µmol of product released per minute per mg of protein; U/mL). The protein concentration in the crude extracts was determined using the Bradford method with bovine serum albumin as the standard.

### Statistical analysis

All data are presented as the mean ± standard error (SE) of three biological replicates. Statistical analysis was performed using SPSS software (Version 19.0), followed by Duncan’s multiple range test for mean separation. Differences were considered statistically significant at *p* < 0.05.

## Results

### Spatial-temporal dynamics of CWDE activities during *A. alternata* P1-1W infection

**Figure 1:**
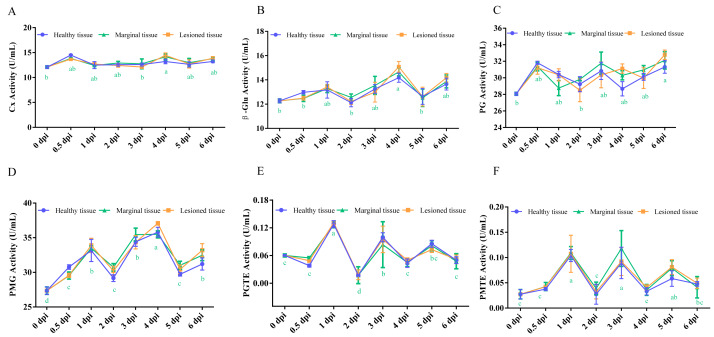
Comparison of the activities of (A) Cx, (B) β-Glu, (C) PG, (D) PMG, (E) PGTE and (F) PMTE among the different tissues during *A. alternata* P1-1W on kiwifruit. The letter at the line indicates significant difference at the different infection times at disease-health junction (*P* < 0.05).

To dissect the spatial dynamics of infection, we comparatively analyzed the activities of CWDEs in healthy tissue, marginal tissue, and lesioned tissue following inoculation with *A. alternata* P1-1W. All six enzymes exhibited broadly similar temporal trends across the three tissue zones (Fig. 1). No statistically significant differences in enzyme activity were observed among tissue types. In contrast, time post-inoculation exerted a highly significant effect (*p* < 0.001) on the activity of each CWDE (Table S1), indicating that infection duration rather than spatial location is the primary determinant of enzymatic changes.

A more detailed examination of marginal tissues revealed distinct, stage-specific kinetic patterns (Fig. 1). For instance, Cx activity showed a mild change over the course of infection, reaching a single maximum at four dpi (14.45 U/mL) (Fig. 1A). Similarly, β-Glu activity peaked at four dpi (14.66 U/mL), representing a 19.49% increase over the control level (Fig. 1B). PG activity increased progressively, peaking at six dpi (32.79 U/mL), a 17% increase relative to baseline (Fig. 1C).

In contrast, PMG exhibited the most rapid and dynamic response. Its activity increased markedly by 0.5 dpi, reached an initial peak at one dpi, transiently declined at two dpi, and then rose again to its absolute maximum (37.08 U/mL) at four dpi (Fig. 1D). This multiphasic pattern underscores the pivotal and early role of PMG in the infection strategy of *A. alternata* P1-1W. Although the activities of PGTE and PMTE showed an early, transient increase and peaked at one dpi, they remained consistently low throughout the time course (Figs. 1E and 1F).

### Spatial-temporal dynamics of CWDE activities during *A. tenuissima* P1-2W infection

Consistent with the observations for *A. alternata* P1-1W, comprehensive analysis of the six CWDEs during *A. tenuissima* P1-2W infection revealed no significant differences in enzyme activities among the three tissue types. In contrast, infection time exerted a significant to highly significant effect on the activity of each enzyme tested (Table S1), confirming that the progression of infection—rather than spatial location—is the primary factor modulating CWDE activity in *A. tenuissima* P1-2W, a pattern also observed in *A. alternata* P1-1W.

**Figure 2:**
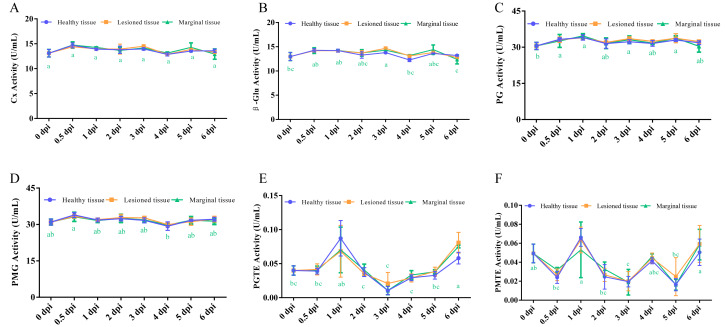
Comparison of the activities of (A) Cx, (B) β-Glu, (C) PG, (D) PMG, (E) PGTE and (F) PMTE among the different tissues during *A. tenuissima* P1-2W on kiwifruit. The letter at the line indicates significant difference at the different infection times at disease-health junction (*P* < 0.05).

Compared with *A. alternata* P1-1W, the enzyme activities in the marginal zone of *A. tenuissima* P1-2W exhibited relatively weak variation over time (Fig. 2). Specifically, Cx activity remained stable throughout the infection period, with no significant temporal changes (Fig. 2A). β-Glu, PG, and PMG activities showed only minor fluctuations and no statistically significant differences over the course of infection (Figs. 2B–2D). PGTE and PMTE activities increased at one dpi and then decreased, reaching another peak at six dpi, but they remained consistently low (<0.10 U/mL) across all time points (Figs. 2E and 2F). Collectively, these findings indicate that the enzymatic alterations induced by *A. tenuissima* P1-2W at the host–pathogen interface were notably more subdued and less dynamic than those triggered by *A. alternata* P1-1W.

### Spatial-temporal dynamics of CWDE activities in control fruits

**Figure 3:**
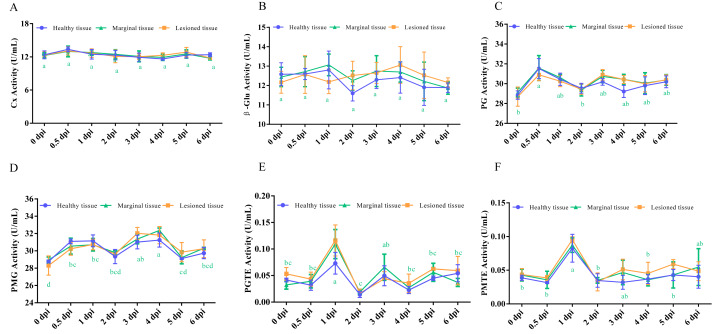
Comparison of the activities of (A) Cx, (B) β -Glu, (C) PG, (D) PMG, (E) PGTE and (F) PMTE among the different tissues in control kiwifruit. The letter at the line indicates significant difference at the different infection times at disease-health junction (*P* < 0.05).

To eliminate the potential influence of kiwifruit-derived CWDEs, we assessed the activities of the same enzymes in control fruit at corresponding time points. Overall, the enzyme activities in control fruit were consistently lower than those in the two fungal treatments throughout the experimental period. Our results showed that both Cx and β-Glu activities remained relatively stable over time, with no significant temporal fluctuations (Figs. 3A, 3B). PG activity peaked at 0.5 dpi, after which no statistically significant changes were observed (Fig. 3C), a pattern similar to that of *A. tenuissima* P1-2W. PMG activity exhibited two distinct phases of increase: the first occurred at 0.5 dpi (30.54 U/mL) and one dpi (30.71 U/mL), while the second culminated in a peak at four dpi (32.36 U/mL) (Fig. 3D). In contrast, the activities of PGTE and PMTE remained consistently low across all time points, with only a transient increase at one dpi (Figs. 3E, 3F). Taken together, these findings indicate that although the six enzyme activities in control fruit displayed broadly similar temporal patterns, notable differences in their dynamics were also evident at equivalent time points.

### Dynamics of CWDE activities during *A. alternata* P1-1W infection

**Figure 4:**
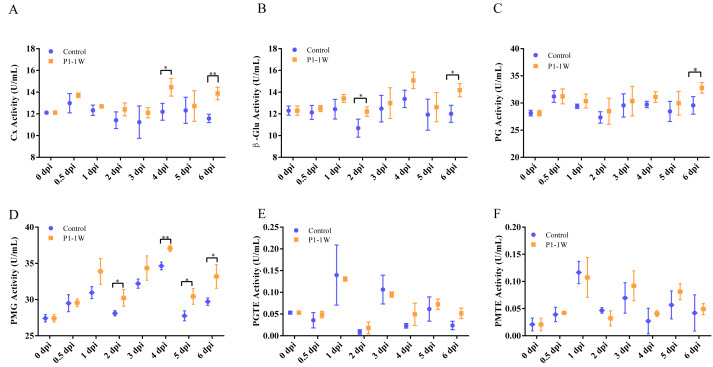
The activities of (A) Cx, (B) β-Glu, (C) PG, (D) PMG, (E) PGTE and (F) PMTE in different infection periods of *A. alternata* P1-1W on kiwifruit. The asterisks indicate significant difference between control and *A. lternata* P1-1W -inoculated fruit (* *P* < 0.05, ** *P* < 0.01, *** *P* < 0.001).

To further characterize the CWDE profile associated with *A. alternata* P1-1W pathogenesis, we compared the activities of six enzymes over the course of infection with mock-inoculated controls. Because no significant differences were detected among the three tissue types examined, enzyme activities were assessed using marginal tissues. Our results showed that fruit inoculated with *A. alternata* P1-1W exhibited significantly elevated activities in four of the six enzymes analyzed (Fig. 4). Cx activity increased progressively, peaking at four dpi (14.45 U/mL), which was a 19.32% increase over the control level (Fig. 4A). Activity then declined at five dpi before increasing again significantly at six dpi relative to the control. β-Glu activity also reached its maximum (15.08 U/mL) at four dpi, with no significant difference compared to the control (Fig. 4B). However, β-Glu activity exhibited an earlier response than Cx, with a significant increase detectable by two dpi compared to the control. Additionally, β-Glu activity was also significantly elevated at six dpi.

PG activity followed a temporal pattern similar to that of the control, with a significant difference observed only at six dpi, when it reached its highest level (32.79 U/mL) (Fig. 4C). Notably, PMG exhibited the most rapid and sustained induction among the enzymes examined, exceeding control levels as early as one dpi. Although PMG activity showed two declines at two and five dpi, it remained significantly higher than in control fruit throughout the experimental period. Peak activity (37.08 U/mL) was recorded at four dpi, representing the highest level among all measured enzymes (Fig. 4D). In contrast, the activities of PGTE and PMTE remained consistently low throughout infection and did not differ significantly from the control at any time point (Figs. 4E, 4F).

To evaluate the relative contribution of each enzyme to pathogenicity, we analyzed the frequency of statistically significant differences between infected and control tissues across all time points (Table S2). PMG displayed the highest significance frequency (57.14%), being elevated in infected fruit at most sampling times, which underscores its central role in the infection process. Although β-Glu and Cx showed a lower significance frequency (28.57%), its marked induction at specific stages suggests a potent, phase-dependent function. The roles of PG appeared more constrained, with a significance frequency of 14.29%. Consistent with their low activity, no significant differences were detected for PGTE or PMTE, confirming their minimal contribution to *A. alternata* P1-1W pathogenicity under these experimental conditions. Collectively, these data delineate the key enzymatic components of *A. alternata* P1-1W’s strategy for host tissue degradation. These findings suggest that this strain employs a more balanced enzymatic strategy during the early stages of infection, characterized by the coordinated involvement of PMG and β-Glu.

### Dynamics of cell wall-degrading enzyme activities during *A. tenuissima* P1-2W infection

**Figure 5:**
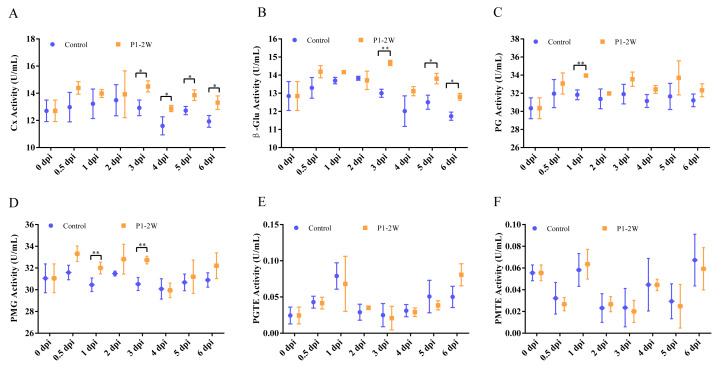
The activities of (A) Cx, (B) β-Glu, (C) PG, (D) PMG, (E) PGTE and (F) PMTE in different infection periods of *A. tenuissima* P1-2W on kiwifruit. The asterisks indicate significant difference between control and *A. tenuissima* P1-2W -inoculated fruit (* *P* < 0.05, ** *P* < 0.01, *** *P* < 0.001).

A parallel analysis of CWDE activities during infection by *A. tenuissima* P1-2W revealed a distinct kinetic profile (Fig. 5). Cx activity peaked at three dpi (14.50 U/mL), declined at four dpi, and then increased again at five and six dpi, with all four time points showing significantly higher activity than the control (Fig. 5A). Similarly, β-Glu activity followed a temporal pattern comparable to that of Cx, but exhibited a more pronounced difference from the control at three dpi (*p* < 0.01). No significant difference was observed at four dpi (Fig. 5B). PG activity in inoculated fruit peaked at one dpi (33.96 U/mL), significantly exceeding that of the control (Fig. 5C). This early PG response in *A. tenuissima* P1-2W contrasted with the late response observed in *A. alternata* P1-1W. PMG activity also showed an early response, becoming significantly different from the control by two dpi and remaining elevated at three dpi (32.73 U/mL). Although PMG activity reached its highest level at two dpi (32.82 U/mL), this value did not differ significantly from the control (Fig. 5D). Consistent with observations for *A. alternata*, the activities of PGTE and PMTE again remained minimal throughout the infection course (both < 0.10 U/mL) (Figs. 5E, 5F).

Statistical evaluation of the activity profiles highlighted clear differences in the enzymatic strategy of *A. tenuissima* P1-2W compared to *A. alternata* P1-1W (Table S2). For *A. tenuissima*, Cx showed the highest significance frequency (57.14%), followed by β-Glu (42.86%), identifying these cellulolytic enzymes as the dominant pathogenic factors. PMG (28.57%) and PG (14.29%) displayed lower significance frequencies, indicating secondary roles. PGTE and PMTE confirmed no significant involvement in pathogenicity. Collectively, these results suggest that *A. tenuissima* P1-2W primarily employs cellulolytic enzymes (Cx and β-Glu) as the main drivers of tissue degradation, while pectinolytic enzymes (PMG and PG) contribute during the early stages of infection.

### Comparative analysis of key CWDE activities between *A. alternata* P1-1W and *A. tenuissima* P1-2W

**Figure 6:**
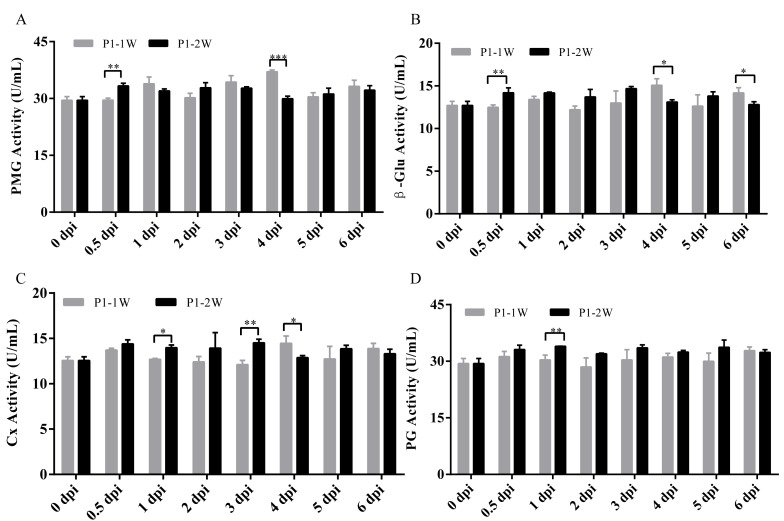
Comparison of the activities of (A) PMG (B) PG (C) Cx (D) β- Glu between *A . alternata* P1-1W and *A. tenuissima* P1-2W on kiwifruit. The asterisks indicate significant difference between two *Alternaria* species at each infection time (* *P* < 0.05, ** *P* < 0.01, *** *P* < 0.001).

Based on the above analyses, four enzymes—PMG, PG, β-Glu, and Cx—were identified as being actively modulated during infection by both *Alternaria species*. A direct, time-resolved comparison of these key CWDEs revealed distinct inter-species divergence patterns across three temporal phases (Fig. 6). The first phase of divergence occurred during the early response at 0.5 and one dpi. Specifically, PMG and β-Glu activities differed between the two strains as early as 0.5 dpi (Figs. 6A, 6B), whereas Cx and PG showed interspecies differences beginning at one dpi (Figs. 6C, 6D). These results indicate that the four enzymes contribute differentially during the initial stages of infection by the two *Alternaria* species. The second phase of divergence was observed around peak infection (3–4 dpi). Differences in PMG and β-Glu activities emerged at three dpi (Figs. 6A, 6B), while Cx activity diverged at both three and four dpi (Fig. 6C). The third and final phase of divergence was detected at six dpi, where PG activity exhibited a marked interspecies difference (Fig. 6D). Taken together, these results revealed that the key pathogenic enzymes exhibited distinct temporal dynamics, highlighting divergent pathogenic strategies between two *Alternaria* species.

## Discussion

Traditional views have long held that fungal pathogenicity primarily hinges upon the release of toxins or the rapid disintegration of host tissues, facilitating immediate exploitation of plant resources (Ma et al., 2019; Tsuge et al., 2013). A previous study from our group has revealed that pectinases and cellulases are the important pathogenic factors in kiwifruit rot soft (Ling et al., 2024a). In this study, our spatiotemporal analysis reveals a more nuanced picture: infection time—rather than the spatial distribution of lesions—emerges as the principal determinant of CWDE activity patterns. This aligns with a developmentally programmed mode of pathogen regulation, wherein fungi meticulously orchestrate their enzymatic repertoire through tightly timed gene expression sequences (Lu et al., 2025; Sánchez-Torres, González-Candelas & Ballester, 2024). Such a strategy ensures precise adaptation to the dynamic changes in host tissue, rather than relying on passive enzyme release from aging or disintegrating tissue (Binyamin et al., 2019).

Our results identified two pectinases (PMG and PG) and two cellulases (Cx and β-Glu) as the primary enzymatic contributors to infection for both *Alternaria* species, whereas the activities of PGTE and PMTE were negligible. This result was consistent with the findings for *Diaporthe* species (Ling et al., 2024a). Despite sharing infection time-based regulation, the two *Alternaria* species deploy fundamentally distinct CWDE strategies. *A. alternata* P3-1W employs a PMG-centric, biphasic strategy: PMG is induced by one dpi, peaks at four dpi, and prioritizes sustained pectin degradation. This pectinase acts in concert with other CWDEs—such as PG and two cellulases—to collectively dismantle the plant’s physical defence barriers. Many studies have established PMG as an indispensable core pathogenicity factor in numerous plant pathogens that can cause various important diseases, including kiwifruit soft rot (Zhang et al., 2025), citrus (Ma et al., 2019), tomato (Wang et al., 2025), and postharvest pepper rot (Li et al., 2024). Based on an in-depth understanding of the mechanism of PMG action, researchers have developed innovative disease control technologies—such as smart nanopesticides that respond to pectinase activity—enabling precise, efficient, and green management of kiwifruit soft rot (Li et al., 2025; Mo et al., 2026).

Conversely, *A. tenuissima* adopts a cellulase-dominant strategy: Cx and β-Glu exhibit sustained mid- to late-elevation (3–6 dpi), driving tissue maceration *via* cellulose hydrolysis. Pectinases serve transiently as an “advance guard” (PG at one dpi, PMG at 2–3 dpi). In plant–pathogenic fungus interactions, disease progression is closely associated with the activities of CWDEs such as PG and Cx (Bhardwaj et al., 2022). The actions of these enzymes are not isolated; rather, their corresponding gene expression exhibits distinct temporal patterns, supporting the hypothesis of a “temporal division of labor” during infection: the early stage prioritizes pectin degradation to open invasion channels, while the later stage may focus on decomposing structural components such as cellulose to promote lesion expansion. Plants are not passive victims; they have evolved corresponding defense proteins, such as polygalacturonase-inhibiting proteins (PGIPs), which can specifically recognize and inhibit the PG activity of pathogenic fungi, thereby delaying lesion expansion (Bhardwaj et al., 2022; Ingel et al., 2019). Furthermore, pathogens may evade PGIP inhibition by secreting multiple isozymes or altering enzyme characteristics. For instance, *Botrytis cinerea* possesses multiple PG genes (*e.g.*, *BcPG*1–*BcPG*6), which differ in their functions during pathogenesis and their susceptibility to inhibition (Joubert et al., 2007). Elucidating the temporal dynamics of these enzyme activities and their molecular regulatory mechanisms is of great significance for understanding disease development patterns and developing novel resistance strategies, such as breeding crops that express specific PGIPs. Therefore, this diversity of isozymes may represent a strategy employed by pathogens to counteract plant defense, warranting further investigation.

### Conclusion

This study reveals that infection time, rather than spatial lesion progression, governs CWDE activity during kiwifruit soft rot caused by *Alternaria* species, indicating developmentally programmed enzymatic regulation. Critically, *A. alternata* P1-1W and *A. tenuissima* P1-2W employ distinctly different pathogenic strategies: *A. alternata* deploys a PMG-centric approach prioritizing sustained pectin degradation, while *A. tenuissima* adopts a cellulase-dominant strategy where Cx and β-Glu drive tissue maceration, with pectinases contributing only transiently during early invasion. Trans-eliminases (PGTE and PMTE) played negligible roles for both species. These divergent enzymatic strategies have important implications for disease management, suggesting that a deeper understanding of pathogen-specific virulence mechanisms can inform the design of more targeted interventions. Future research should focus on elucidating the molecular regulatory networks that govern these distinct enzyme deployment patterns, thereby facilitating the identification of conserved pathogenic nodes and supporting the development of broad-spectrum strategies for controlling kiwifruit soft rot.

## Supplemental Information

10.7717/peerj.21223/supp-1 Supplemental Information 1 Schematic diagram of zone sampling

10.7717/peerj.21223/supp-2 Supplemental Information 2 Analysis results of the main effects of different spatial zones and infection time points in two *Alternaria* strains

10.7717/peerj.21223/supp-3 Supplemental Information 3 Significant difference frequency of each enzyme between control and inoculated tissue in two *Alternaria* species

10.7717/peerj.21223/supp-4 Supplemental Information 4 Raw data for Figs. 1–5

## Additional Information and Declarations

### Data Availability

The following information was supplied regarding data availability: The raw data is available in the Supplemental File.
