# Supplementary figures and images for: Differential spatiotemporal dynamics of cell wall-degrading enzymes underlie pathogenicity variation in two Alternaria species causing kiwifruit soft rot
Source: PeerJ. 2026 Apr 28;14:e21223. doi: 10.7717/peerj.21223 (PMC13134541; doi:10.7717/peerj.21223)

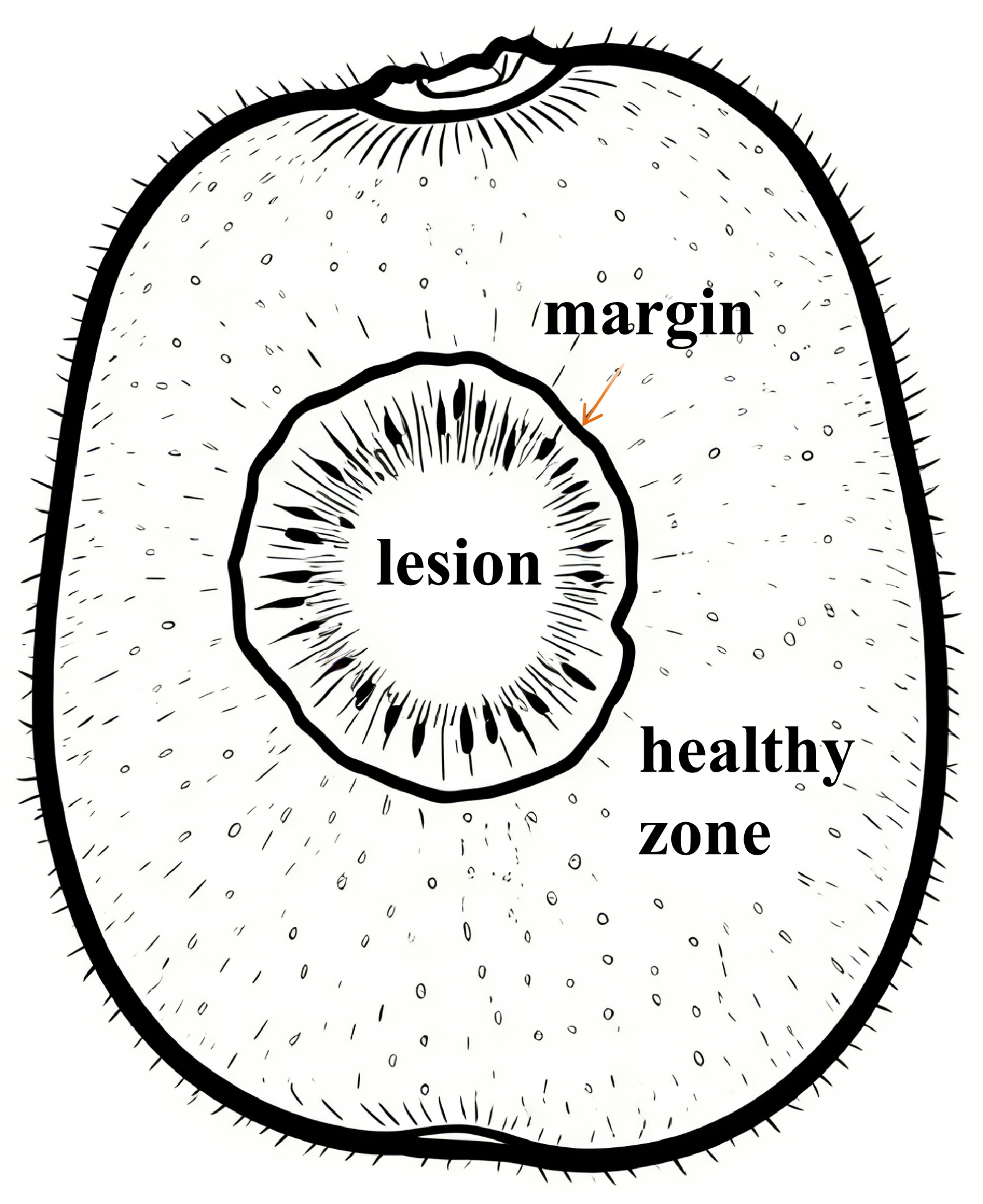

Supplement: Supplemental Information 1 [file peerj-14-21223-s001.png]
