# Supplementary material for: Differential spatiotemporal dynamics of cell wall-degrading enzymes underlie pathogenicity variation in two Alternaria species causing kiwifruit soft rot
Source: PeerJ. 2026 Apr 28;14:e21223. doi: 10.7717/peerj.21223 (PMC13134541; doi:10.7717/peerj.21223)
Supplement: Supplemental Information 2 [file peerj-14-21223-s002.docx]

Table S1 Analysis results of the main effects of different spatial zones and infection time points in two *Alternaria* strains

| Strain | Enzyme | Source | Type III Sum of Squares | Mean Square | F | Sig. |
| --- | --- | --- | --- | --- | --- | --- |
| P1-1W | Cx | spatial location | 0.468 | 0.234 | 0.374 | 0.690 |
|  |  | infection time | 33.999 | 4.857 | 7.776 | 0.000 |
|  | β-Glu | spatial location | 0.229 | 0.115 | 0.181 | 0.835 |
|  |  | infection time | 43.386 | 6.198 | 9.786 | 0.000 |
|  | PG | spatial location | 1.441 | 0.721 | 0.359 | 0.700 |
|  |  | infection time | 103.546 | 14.792 | 7.366 | 0.000 |
|  | PMG | spatial location | 4.534 | 2.267 | 1.489 | 0.236 |
|  |  | infection time | 528.315 | 75.474 | 49.569 | 0.000 |
|  | PGTE | spatial location | 0.000 | 0.000 | 0.037 | 0.964 |
|  |  | infection time | 0.074 | 0.011 | 44.770 | 0.000 |
|  | PMTE | spatial location | 0.001 | 0.000 | 1.467 | 0.241 |
|  |  | infection time | 0.061 | 0.009 | 29.358 | 0.000 |
| P1-2W | Cx | spatial location | 0.118 | 0.059 | 0.067 | 0.935 |
|  |  | infection time | 20.995 | 2.999 | 3.426 | 0.005 |
|  | β-Glu | spatial location | 0.815 | 0.408 | 0.519 | 0.598 |
|  |  | infection time | 25.826 | 3.689 | 4.698 | 0.000 |
|  | PG | spatial location | 2.878 | 1.439 | 0.781 | 0.464 |
|  |  | infection time | 91.120 | 13.017 | 7.066 | 0.000 |
|  | PMG | spatial location | 0.893 | 0.447 | 0.342 | 0.712 |
|  |  | infection time | 78.085 | 11.155 | 8.533 | 0.000 |
|  | PGTE | spatial location | 0.000 | 0.000 | 0.202 | 0.818 |
|  |  | infection time | 0.026 | 0.004 | 20.231 | 0.000 |
|  | PMTE | spatial location | 0.000 | 0.000 | 0.347 | 0.709 |
|  |  | infection time | 0.017 | 0.002 | 17.212 | 0.000 |
