# Supplementary material for: Differential spatiotemporal dynamics of cell wall-degrading enzymes underlie pathogenicity variation in two Alternaria species causing kiwifruit soft rot
Source: PeerJ. 2026 Apr 28;14:e21223. doi: 10.7717/peerj.21223 (PMC13134541; doi:10.7717/peerj.21223)
Supplement: Supplemental Information 3 [file peerj-14-21223-s003.docx]

Table S1 Significant difference frequency of each enzyme between control and inculocated tissue in two *Alternaria* species

| Species | Enzyme | Significant Difference Frequency |
| --- | --- | --- |
| *A. alternata* P1 1W | Cx | 28.57% |
|  | β-Glu | 28.57% |
|  | PG | 14.29% |
|  | PMG | 57.14% |
|  | PGTE | 0.00% |
|  | PMTE | 0.00% |
| *A. tenuissima* P1 2W | Cx | 57.14% |
|  | β-Glu | 42.86% |
|  | PG | 14.29% |
|  | PMG | 28.57% |
|  | PGTE | 0.00% |
|  | PMTE | 0.00% |
